# Supplementary figures and images for: Simplified molecular diagnosis of visceral leishmaniasis: Laboratory evaluation of miniature direct-on-blood PCR nucleic acid lateral flow immunoassay
Source: PLoS Negl Trop Dis. 2024 May 7;18(5):e0011637. doi: 10.1371/journal.pntd.0011637 (PMC11075898; doi:10.1371/journal.pntd.0011637)

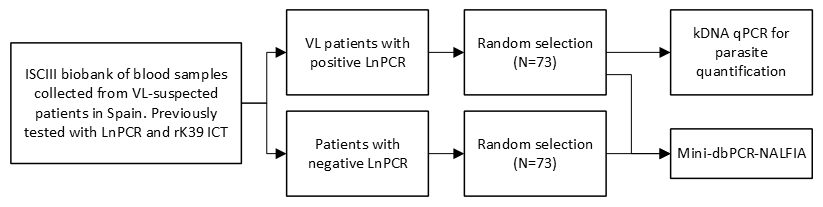

Supplement: S1 Fig — (TIF) [file pntd.0011637.s001.tif]

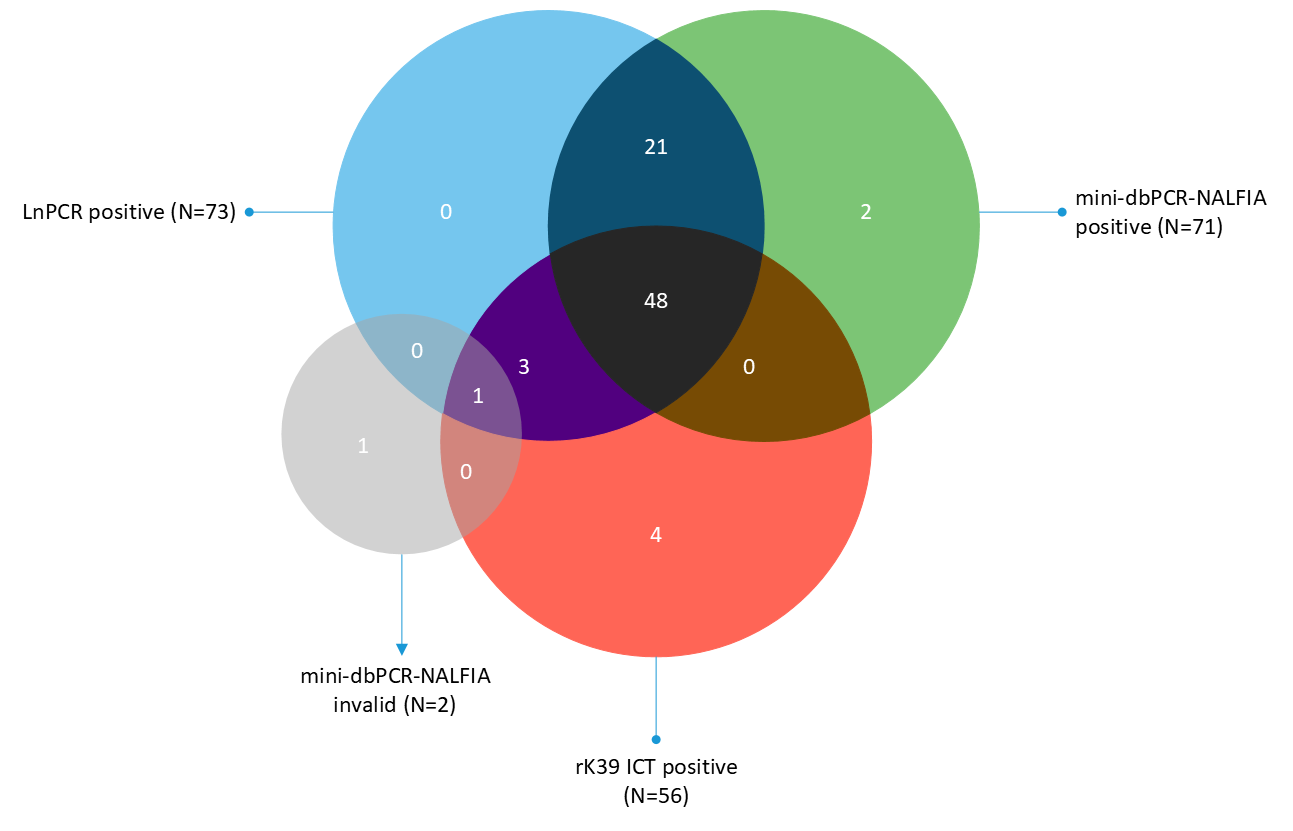

Supplement: S3 Fig — (TIF) [file pntd.0011637.s003.tif]

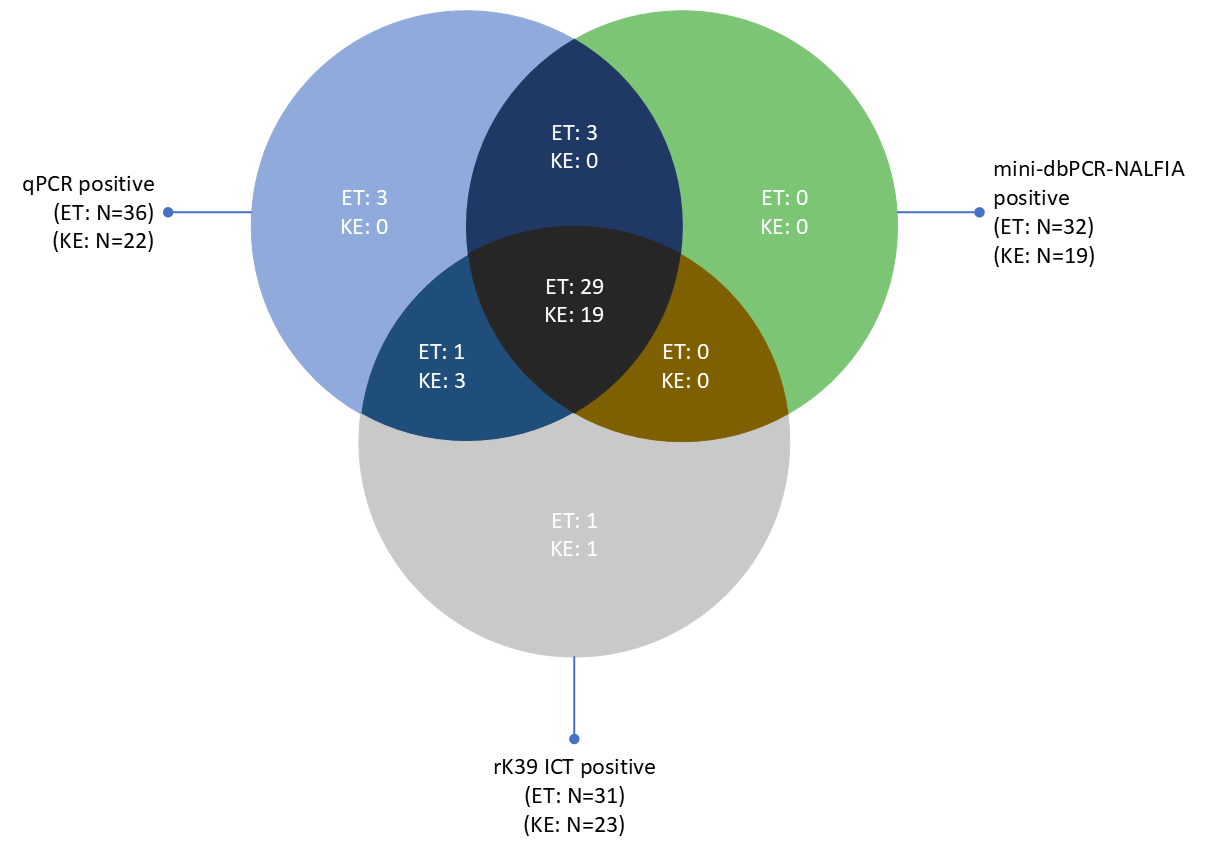

Supplement: S4 Fig — ET: Ethiopia; KE: Kenya. (TIF) [file pntd.0011637.s004.tif]
