# Supplementary material for: Simplified molecular diagnosis of visceral leishmaniasis: Laboratory evaluation of miniature direct-on-blood PCR nucleic acid lateral flow immunoassay
Source: PLoS Negl Trop Dis. 2024 May 7;18(5):e0011637. doi: 10.1371/journal.pntd.0011637 (PMC11075898; doi:10.1371/journal.pntd.0011637)
Supplement: Attachment — Submitted filename: Response to peer review_VL miniPCR_20240123.pdf [file pntd.0011637.s007.pdf]

Response to reviewers manuscript “Mini-dbPCR-NALFIA for diagnosis of VL”

Dear editor,

Please find below the authors’ point-to-point responses to the comments raised by the reviewers. All references to lines in the responses refer to the manuscript with tracked changes.

Kind regards,

Norbert van Dijk (on behalf of all authors)

Methods

<u>Reviewer #1:</u>

Comment reviewer: Yes, though I have a query regarding 5' mismatches in the primers used, which may explain differences in sensitivity in Spain vs. East Africa. The query is within the manuscript. → For many L. donovani sequences in GenBank, there is a mismatches in these primer at the 5’ end. Could this perhaps explain the lower sensitivity in the East African samples? I’ll paste screenshot.
Response authors: The authors thank the reviewer for pointing this out and will consider revision of the primer sequence in further optimization of the assay. The primer sequences of the kDNA qPCR at AMC were actually the same as for dbPCR, so it is unlikely that potential mismatches caused discrepant results for these two assays (and thereby the lower sensitivity).

Comment reviewer: Title suggests that this study developed the assay. But here (line 29-30) it seems it had already been developed and that this study is just evaluating it. If it was developed in this study, abstract should be reframed and methodology should include how it was developed and more details on the assay.
Response authors: The assay has been developed before for malaria (see: PMC10024383) and was adapted for *Leishmania* detection. The manuscript is focused on the evaluation, so references to the development have now been removed throughout the document.

Comment reviewer: Line 156: “Its principle is described in detail elsewhere (28)”. As this is so central to this paper, the principles should be summarised here as well.
Response authors: This particular sentence has been removed (also after comments of reviewer 2). We hope that the principle and separate steps of mini-dbPCR-NALFIA are now addressed in sufficient detail in the introduction and methods.

<u>Reviewer #2:</u>

ABSTRACT

- **Comment reviewer:** In the overall rationale and aim of the study, I think it is important to highlight the invasiveness of the tissue aspirate microscopy, for which the dbPCR-NALFIA could be a solution, rather than focusing on the POC test.
  Response authors: The background of the abstract has been rewritten to better reflect the potential use of mini-dbPCR-NALFIA.
- **Comment reviewer:** Some sections of the abstract can be shortened and instead the diagnostic accuracy of the rK39 RDTs (brand specified) can be added. This especially when authors would still conclude from their study that their test can replace the rK39 RDT.

Response authors: The authors have followed this suggestion and rewritten the abstract accordingly

AUTHOR SUMMARY

- **Comment reviewer:** Line 51: Also in the Americas.
  Response authors: This sentence has been removed to shorten the summary.
- **Comment reviewer:** Line 52: What do the authors mean by "optimum treatment outcomes", a good prognosis?
  Response authors: The authors agree that this sentence was unclear, it has been replaced by "adequate patient management" (line 63-64)
- **Comment reviwer:** Lines 57-59: I do not really think that 500 par/mL is a very low parasitemia, especially for TOC/treatment follow-up you would want a test that can see whether all parasites are cleared from the blood or not.
  Response authors: The authors agree that for a test of cure, a lower limit of detection will be required. However, we believe that this LoD is sufficient to detect the majority of clinical, untreated VL infections. The sentence has been rewritten to better reflect this. (line 69-70)

INTRODUCTION

General remarks:

- **Comment reviewer:** The background section is rather long and contains information that is not necessarily important to understand the content of the manuscript. For example, the authors explain in depth the impact of the disease in East-Africa, then again in the Mediterranean and then focus on outbreaks in southern Europe (nothing about Indian subcontinent?). Please try to reduce this part. Most important is that there is two main causative species in different parts of the world and that due to the elimination initiative in the Indian subcontinent majority of cases are now reported from East-Africa, where available diagnostics have major limitations.
  Response authors: The authors thank the reviewer for this suggestion to improve the introduction. This section has been revised and shortened.
- **Comment reviewer:** Authors mention in Line 84 that the gold standard for VL diagnosis is demonstration of Leishmania parasites in tissue aspirates through microscopy, however, this is only the case in resource-limited countries. I also do not completely agree that this is the gold standard, as it is only the last tool that will be employed if the other available methods in the diagnostic algorithm fail to detect Leishmania infection. Please revise.
  Response authors: see below.
- **Comment reviewer:** Related to the comments above, the section about tissue aspiration is too lengthy and needs to be reduced. In line 89, The authors state that tissue microscopy is unsuited for rural settings, however, in general because of the invasiveness and low sensitivity of the method it is not a preferable approach.
  Response authors: please see below.
- **Comment reviewer:** Overall I think the background would benefit from a shift in the structure, following the diagnostic algorithm.
  Response authors: The authors thank the reviewer for this suggestion to improve the background on currently used diagnostics. The second paragraph has been rewritten and shortened, first discussing rK39 as primary diagnostic, then mentioning its limitations and ending with the lack of a feasible ToC, as neither rK39 nor microscopy are suited.
- **Comment reviewer:** An important advantage of the use of molecular tools is that it can be employed on less-invasive samples like blood.
  Response authors: This advantage has been added (line 132).
- **Comment reviewer:** The authors mention that molecular methods are not employed yet with the focus on East-Africa, however, to my knowledge it is also not routinely employed in the Americas or Indian subcontinent. The authors should either broaden the scope or

highlight that the rK39 RDT diagnostic accuracy is suboptimal in East-Africa and therefore diagnosis often relies on tissue aspiration which does not allow for decentralization of diagnosis and care.
Response authors: The scope of this sentence has been broadened as suggested (line 137)

- **Comment reviewer:** The authors could still highlight that in Sudan, it was recommended to incorporate LAMP in the diagnostic algorithm for VL, before tissue microscopy would be done. However, major limitations to do so are indeed the mentioned disadvantages, but I think also still the cost of a LAMP assay.
Response authors: The limitation of the costs has been added to this paragraph (line 150-151). To avoid that the paragraph becomes too lengthy, the example of Sudan has not been added.
- **Comment reviewer:** In line 126, can the authors specify that one target is to check for DNA extraction efficiency and indicate which Leishmania marker is targeted? kDNA? If so, maybe highlight why kDNA was selected as target.
Response authors: The authors have added this information in the Methods section (line 201-202) as it felt more appropriate there.
- **Comment reviewer:** Perhaps good to briefly highlight the diagnostic accuracy found so far of the mini-dbPCR-NALFIA, and then proceed with the rationale of the study, which is not clearly described now. Furthermore, from the overall objective it is currently not clear what the reference test was. A composite reference of rK39 + qPCR or nPCR? This is not clear, please clarify.
Response authors: The objective of the study has been reformulated. As this is the first study to evaluate the mini-dbPCR-NALFIA for *Leishmania* diagnosis, no previous diagnostic accuracy can be reported. The assay has been evaluated before for *Plasmodium* (see: PMC10024383) but since this concerns a different pathogen with different PCR targets, it is not representative of the accuracy that may be expected for the *Leishmania* assay.

METHODS
Ethics
Comment reviewer: Samples have been exported out of East-Africa for analysis in the Netherlands. Can the authors specify whether (national?) IRBs approved this and indicate whether there was an MTA allowing this?
Response authors: Specifications on the export of the East African samples to the Netherlands have been added.

General comments

- **Comment reviewer:** More information needs to be provided on the source of the samples/study population. Was it secondary use of biobanked samples or fresh samples? If the former, did patients consent to the use of their samples for secondary research? What did the patient population look like? What was used as a definition of VL? Was any sample size calculation performed? Please elaborate futher on study population and sample collection/storage in a separate paragraph.
Response authors: A separate section on the used sample sets has been added, addressing the points mentioned by the reviewer. The patient consent for sample use has been added to the ethical clearance section. Sample size calculations are described in lines 253 – 258.
- **Comment reviewer:** It seems like part of the methods section is missing after line 162. What were the reaction conditions used?
Response authors: During submission of the manuscript, the original submitted word document was erroneously altered by the submission portal software: part of the method section was removed and inserted later in the manuscript. Unfortunately, the authors only noticed this when the manuscript had already been send out for review. The revised

document does not contain this error, and the reaction conditions are described in lines 213 - 215

- **Comment reviewer:** Can the authors explain why they did not go lower than 10 par/mL for the LoD? The LoD of kDNA is known to be much lower than that.
  Response authors: dbPCR is performed directly on 2.5 µL of blood, without DNA concentration step. This means that 10^0 p/mL equals 0.0025 p/reaction, a very low density where the chance of stochastic variation between tests becomes significant. 10^0 p/mL had been tested in an earlier phase and consistently gave negative results with NALFIA. This concentration was therefore not included in the 20 repeats for the determination of the LoD.
- **Comment reviewer:** Why are the results of the rK39 RDT already mentioned in the methods if this was a study procedure and so a result?
  Response authors: The method section on the sens/spec evaluation has been rewritten to clarify that all samples used in this study had already been tested with rK39 ICT previously, at the time of collection. Hence, rK39 testing was not a study procedure, these results were already available and were used for the selection of Kenyan and Ethiopian samples for this study.
- **Comment reviewer:** Perhaps it would help to have a flow chart or table in the methods to clarify the sample sets used for testing: where samples came from (especially in East-Africa: it is strange it comes up in the last sentence only and is still unclear), what was considered a VL-suspected patient, whether it was a prospective collection on secondary use of samples (in case of the latter, how were samples selected considering that authors knew which ones were – strongly – positive and selected exactly half negatives/positives), how they were tested previously/for the study etc. Going through the text, it is not clearly described and numbers do not seem to add up but that is perhaps due to the fact that the way it is described is a bit confusing. It would be useful if the authors considerably revise this section and add a figure or table to guide it.
  Response authors: We thank the reviewer for pointing out that this description was not clear and for the suggestion of a supporting figure as well. The section on the sensitivity/specificity evaluation has been rewritten to clarify where the samples originated from, how they were selected and which procedures were done previously and which specifically for this study. Two supplementary figures (S1 and S2) were made to visualize this.
- **Comment reviewer:** Can the authors clarify the cut-off of 36 they used for positivity of the kDNA PCR or refer to a paper that describes this?
  Response authors: This cut-off has been based on in-house (unpublished) validation of the kDNA qPCR and renders the most accurate results in terms of sensitivity and specificity. This was added to the manuscript (line 362)

Results

<u>Reviewer #1:</u>
Comment reviewer: [reviewer made a comment about discrepant numbers in Table 2.]

Response authors: To comment on the discrepant numbers in Table 2: the authors thank the reviewer for noticing this. This was because 2 samples from Spain had an invalid NALFIA result and were excluded from the NALFIA comparison with LnPCR, but not for the rK39 ICT – LnPCR comparison. This has now been indicated in table 2.

Comment reviewer: Table 3: Could it be that scoring of qPCR thresholds may have included false positives. Were denaturation curves run to verify qPCR products? Alternatively, could the mismatches in the strip assay have limited detection in the East African context?

Response authors: No denaturation curves were run to verify the specificity of the PCR products, meaning that we cannot exclude the occurrence of some false-positive results in qPCR. This was added to the discussion (line 594-595). However, we consider the cut-off of 36 relatively conservative, and based on in-house validation of the kDNA qPCR at AMC, false-positive signals with a Cq <36 are deemed unlikely. The reliability of the qPCR results is also supported by the sensitivity estimates for rK39 ICT in East Africa which match with literature reports (see revised discussion). Regarding the mismatch hypothesis, please refer to our comment above.

Reviewer #2:

RESULTS

- **Comment reviewer:** It is not entirely clear to me how the authors calculated with this much depth the LoD of the mini-dbPCR-NALFIA that the difference between 500 and 650 par/mL can be detected.
  Response authors: To get a first idea of the LoD, only 10-fold dilutions were tested. If all 20 tests were positive for a given concentration, but the majority of tests for one concentration lower was negative, an intermediate concentration between these two was tested to estimate the LoD with more precision. For example, for *L. donovani* all 20 tests were positive for 1000, but for 100 and 500 p/mL, 19/20 tests were negative. Therefore, intermediate concentrations of 750 and 650 p/mL were tested to determine the LoD in more depth. To clarify this approach, a sentence was added to the LoD methods (lines 242-243)
- **Comment reviewer:** Line 385: were these samples that were mini-dbPCR-NALFIA positive also positive by kDNA PCR?
  Response authors: As LnPCR was the reference test for the samples from Spain, kDNA qPCR was only performed on the LnPCR-positive samples to quantify their parasite densities, and on a small number of randomly selected LnPCR-negative samples (as negative controls). This subset included one of the two samples that was NALFIA positive: it was negative in the kDNA qPCR, suggesting that it was truly negative and the positive NALFIA result may have been due to contamination.
- **Comment reviewer:** To show the agreement between the different tests, instead of only using values in the text, authors could perhaps add a (supplementary) VENN diagram to show the overlapping results in the three sample sets.
  Response authors: Two Venn diagrams were created to visualise the results for Spain and East Africa (supplementary figures S3 and S4).

FIGURES AND TABLES

- **Comment reviewer:** The authors should ensure that the figures and tables are presented in the text at the right time and should link to it in their text.
  Response authors: The authors have checked the figure and table references in the text and tables were moved and placed directly after the paragraph where they were first mentioned.
- **Comment reviewer:** The authors indicate in figure 1 that a band for kDNA but not for GAPDH is still considered positive. Is that a likely possibility? Could it be contamination is this case?
  Response authors: In principle, the GAPDH line should always be positive, since human GAPDH DNA is present in any human blood sample that is tested. However, due to the high copy number of kDNA, it has been observed that amplification of the GAPDH target can be inhibited by the amplification of the kDNA target in case of high parasite input. Contamination could be a possibility as well, this cannot be distinguished from the scenario explained above.
- **Comment reviewer:** There is no reference in the text to Table 2.
  Response authors: The reference was there in the original manuscript document but was corrupted by the submission system. This has been corrected.

Conclusions

<u>Reviewer #1:</u>

Comment reviewer: Line 365: "The sensitivity of mini-dbPCR-NALFIA in East African samples was found to be slightly lower" But using a different reference test. Could there be limitation of reference tests?
Response authors: The authors agree that the difference may have been affected by the difference in reference test, as is mentioned in the same paragraph. The limitation of using different references has been mentioned in the second last paragraph of the discussion, and the absence of a melting curve analysis has been added there.

<u>Reviewer #2:</u>

- **Comment reviewer:** As throughout the rest of the text, also here the authors can be more comprehensive. The first Alinea of the discussion is repetitive from the introduction and repeats again what the overall aim of the study was. Methods used are explained again and results are repeated. Authors should focus more on how results should be interpreted and placed in a broader context.
  Response authors: The authors acknowledge that the original discussion was too lengthy and have rewritten it to make it more succinct.
- **Comment reviewer:** Authors mention that further optimization could be done to improve the LoD but do not mention what can be done to indicate that indeed there is potential that it could be improved. I was a bit surprised to see that kDNA, which normally has a very low LoD in PCR, resulted in a high LoD. Is this most likely due to the direct PCR on blood without extraction or due to the read-out with the lateral flow? Can the authors reflect on this? Also the word "could" should probably be "should", because with the current diagnostic accuracy I do not see how otherwise it would ever be implemented in routine practice in the field.
  Response authors: The authors agree that further optimization will indeed be necessary to make dbPCR-NALFIA suitable for routine practice. During the development of the assay, high specificity was initially prioritized over a low LoD (due to the burden and toxicity of antimonial treatment used in East Africa, false-positive results should be prevented). Suggestions for (PCR) optimization have been added in line 499-500. The NALFIA read-out may have less potential for optimization, as increasing the volume of PCR product loaded on the strip has so far not resulted in higher sensitivity in our experiments.
- **Comment reviewer:** The authors state: "The lower sensitivity in East Africa may have resulted from parasite densities close to the LoD of mini-dbPCR-NALFIA, found in a number of samples and leading to a false-negative NALFIA result." These data are available from the qPCR right? Can the authors add these data to the results and make the statement stronger?
  Response authors: The parasite densities of the false-negative NALFIA results are mentioned in the result section.
- **Comment reviewer:** It is mentioned that the InBios RDT in Spain had a considerably lower sensitivity than the dbPCR-NALFIA, which was not the case in East-Africa (where IT-Leish is used). Could this be intrinsic to the diagnostic accuracy of the specific RDT brand? Can the authors put this finding in a broader context and compare the obtained sens/spec with the literature. Or is it possible that if the RDT was used on stored samples that its sensitivity decreased as it's normally employed as a POC test? To clarify this, authors should refer more to existing literature.
  Response authors: Throughout the manuscript, the brand difference has been highlighted more, and it has been mentioned in the discussion to explain the difference in performance.

All rK39 ICTs for this study were performed at the time of sample collection, which has been more clearly explained in the revised methods.

- **Comment reviewer:** In the section about the employment of the dbPCR- NALFIA in East-Africa the authors should focus their statement particularly on the test-of-cure and diagnosis of relapsing patients. Except if the sensitivity can greatly be increased, it will probably not be employed in EA. However, for example in northwest Ethiopia, there is a very high proportion of HIV-VL coinfected patients, who still frequently undergo an invesive TOC and are repeatedly relapsing. For such populations, its employment would mostly be beneficial.
  Response authors: We agree with this reflection, the section has been adapted.
- **Comment reviewer:** It would be good if the authors can explain in the manuscript what the cost is of the current test, including instruments needed. In order for the test to be cost-beneficial, also in Spain, it should not only be easy and have good diagnostic accuracy, but also be cheap, unless of course if it would only be used for few patients. This is an important operational challenge that is often not described, but critical information for decision makers.
  Response authors: A paragraph on the costs of mini-dbPCR-NALFIA has been added (line 567-573)
- **Comment reviewer:** The strengths and weaknesses of the study can be written more concise.
  Response authors: This section has been shortened.
- **Comment reviewer:** In the conclusion, rather than using the test for POC diagnosis in place of the rK39 RDT, I think more focus can be put on its use for test-of-cure and relapsing patients, and so to get rid of the highly invasive aspirates for patients. Furthermore, authors can be more careful with their statement about Spain as well if the costs are higher than the lateral flow assays, because then take-up of the dbPCR-NALFIA would probably still not be as a first-line diagnosis, but maybe when the rK39 RDT is negative and there is still high suspicion of VL.
  Response authors: The conclusion was rewritten to emphasize the potential of the assay as test of cure and for relapse diagnosis.
- **Comment reviewer:** Another reflection, however, is that as a test-of-cure one would like to have a very sensitive test, because even blood parasite load does not mean that the patient is completely cleared. So even though the dbPCR-NALFIA is less invasive, its LoD is still not really optimal for TOC and so should be further improved indeed.
  Response authors: We agree with this remark and this has been mentioned in the discussion (line 497-499). As mentioned by the reviewer, mini-dbPCR-NALFIA would already be a significant improvement compared to microscopy, both in terms of sensitivity and sampling invasiveness.
- **Comment reviewer:** The authors describe a couple of times in their text that the test under validation is very robust. However, it did not provide valid results for some of the samples, which is not discussed anymore in the rest of the manuscript, but which indicates that it is not completely robust. Can the authors further elaborate on this and maybe compare this with the validity of other tests employed in routine?
  Response authors: In total, only 2 of the 282 tested samples had an invalid test result, which may have resulted from an error during PCR preparation. This is indeed not 100% robust, but nevertheless very robust, also when considering that the test performed well in different laboratories and in the hands of different operators. Some adaptations have been made in the manuscript, including an added sentence in lines 582-583. No literature has been found on frequency of invalid rK39 ICT results (IT LEISH or Kalazar Detect) in routine practice, hence no comparison could be made here

----

Editorial and Data Presentation Modifications?

Reviewer #1:
Comment reviewer: I have made minor edits and suggestions in the attached track-changes document.
Response authors: These have been processed.

Reviewer #2:

BACKGROUND

Comments reviewer:
- Line 68: Check sentence construction "caused by infection with protozoan Leishmania parasites"
- Line 85: Please change "aspirates of infected tissues" to "tissue aspirates"
- Line 93-96: Try to reduce the length of the sentence, e.g. "They are unsuitable for monitoring of VL treatment response and diagnosis of VL relapse".
- Line 98: "Where VL-HIV co-infection is common". Also no need to mention again that the rK39 sensitivity is significantly lower, that is already mentioned in the previous sentence.
- Line 121: I would recommend to change DNA purification to DNA isolation or extraction
- Line 128: I would advice the authors not to focus too much on the "rural" endemic settings. Maybe they are referring to low-resource, endemic settings? I assume that the test is not appropriate yet to bring to the field during outreach campaigns as is done with the rK39 ICTs.

Response authors: All suggested changes above have been made.

METHODS

- **Comment:** Lines 154-155: I feel this information should be in the background section. The part about he principle is already explained in the background, so can be omitted.
  Response: These sentences have been rewritten and the information has moved to the background
- **Comment:** Line 162: Sentence seems unfinished.
  Response: This was due to the document corruption by the submission portal mentioned earlier. This has been resolved in the revised document.
- **Comment:** 167: Please change negative donor blood to blood from a non-endemic control
  Response: This has been changed.
- **Comment:** 164-167: This sentence is a brief explanation of the rest of the paragraph and can be omitted as it does not add anything extra.
  Response: Agreed.
- **Comment:** 186:187: Perhaps the authors can specify Spain (L. infantum) and East-Africa (L. donovani), to emphasize that both were targeted, which is a strength of the study.
  Response: Thank you for the suggestion, this has been added.
- **Comment:** Authors should mind that a lot of their sentences start with numbers; if this is done it should be written in full.
  Response: This has been checked and corrected throughout the document.

RESULTS

- **Comment:** Please have a look at line 378 and 413 and solve the issue with the insertion of the reference.

Response: This has been corrected (it was again an error introduced by the processing of the manuscript by the submission system)

- **Comment:** Line 417: were these 3 dbPCR-NALFIA positives also the ones that were rK39 positive? Again here a VENN diagram would easily show this I feel.
  Response: There were 2 endemic controls from Ethiopia with a positive rK39. One of these was also qPCR positive; both were NALFIA negative. This has not been added to the text because this would probably make this section too information-dense, but it can be seen in the VENN diagram (S4 Figure).
- **Comment:** Line 419: please revise the sentence "samples from healthy volunteers in non-endemic areas"
  Response: This has been done.
- **Comment:** 422-424: in three separate sentences, it was explained that agreement is excellent/perfect for different tests. Please make more comprehensive and avoid repetitive use of words and sentences.
  Response: This has been rewritten

DISCUSSION

Comments:

- Lines 448 – 449: "Given that the analytical LoD difference for L. infantum and L. donovani is relatively small, the diagnostic detection limit of mini-dbPCR-NALFIA for both species is likely to be comparable." Sentences who do not add much from the text like this one should be omitted. Authors can just mention that the LoD was still rather high, although majority of samples tested had a parasite load above the LoD.
- Lines 461 – 462: "This is consistent with the fact that sensitivity and specificity are inversely related; when sensitivity increases, the specificity tends to decrease, and vice versa (37)." This information is redundant for readers and can be omitted.

Response: This has been changed.

Summary and General Comments

Reviewer #1:
Comment reviewer: This is a well-written manuscripts of well-conducted research, validating a new field-deployable relatively simple diagnostic tool for visceral leishmaniasis. This is very much needed. I have a number of muinor edits and buried in the attached manuscript.
Response authors: The authors thank the reviewer for the positive feedback on the research, and for the useful comments to improve the manuscript.

Reviewer #2:
Comment reviewer: With the current initiatives around VL elimination in different parts of the world, this manuscript describes a very timely and relevant study with a good design. Clarity of the methods can be improved, and overall the text should be more concise. Authors should also make some reflections about at which level this test would actually be feasible to employ, in terms of added diagnostic value, complexity and cost in comparison with the tests that are routinely used. Overall, if such changes/reflections are made, this manuscript deserves to be published.
Response authors: The authors thank the reviewer for the extensive and constructive feedback on the manuscript. This was very helpful, and we have used the raised comments to improve the manuscript. We hope these revisions have made the manuscript fit for publication. Our thanks also for the positive words about the research itself and the dbPCR-NALFIA assay.
