# Supplementary material for: Simplified molecular diagnosis of visceral leishmaniasis: Laboratory evaluation of miniature direct-on-blood PCR nucleic acid lateral flow immunoassay
Source: PLoS Negl Trop Dis. 2024 May 7;18(5):e0011637. doi: 10.1371/journal.pntd.0011637 (PMC11075898; doi:10.1371/journal.pntd.0011637)
Supplement: Attachment — Submitted filename: VL miniPCR manuscript_FULL_20230829-reviewer.docx [file pntd.0011637.s005.docx]

**Full Title: Simplified molecular diagnosis of visceral leishmaniasis: development and laboratory evaluation of miniature direct-on-blood PCR nucleic acid lateral flow immunoassay**

**Short title: Mini-dbPCR-NALFIA for diagnosis of VL**

Norbert J. van Dijk^1,2#^, Dawit Gebreegziabiher Hagos^1,2,3^, Daniela M. Huggins^1^, Eugenia Carrillo^4,5^, Sophia Ajala^1^, Carmen Chicharro^4,5^, David Kiptanui^6^, Jose Carlos Solana^4,5^, Edwin Abner^6^, Dawit Wolday^7^, Henk D.F.H. Schallig^1,2^.

**Affiliations:**

^1^Amsterdam University Medical Centres, Academic Medical Centre (AMC) at the University of Amsterdam, Department of Medical Microbiology and Infection Prevention, Experimental Parasitology, Amsterdam, the Netherlands.

^2^Amsterdam Institute for Infection and Immunity, Infectious Diseases Programme, Amsterdam, the Netherlands

^3^College of Health Sciences, School of Medicine, Department of Medical Microbiology and Immunology, Mekelle University, Mekelle, Ethiopia

^4^ WHO Collaborating Centre for Leishmaniasis, National Center for Microbiology, Instituto de Salud Carlos III, Majadahonda (Madrid), Spain.

^5^Centro de Investigación Biomédica en Red de Enfermedades Infecciosas (CIBERINFEC-ISCIII), Madrid, Spain

^6^Kacheliba Sub-County Hospital, Kacheliba, West Pokot County, Kenya

^7^Department of Biochemistry and Biomedical Sciences, McMaster University, Hamilton, Canada

^#^Corresponding author: n.j.vandijk@amsterdamumc.nl

**Abstract**

**BACKGROUND:** Diagnosis of visceral leishmaniasis (VL) in resource-limited endemic regions is currently based on serological testing with rK39 immunochromatographic tests (ICTs). However, rK39 ICT frequently has suboptimal diagnostic accuracy and is unsuited for treatment monitoring and detection of VL relapses. In search for more reliable assays for point-of-care VL diagnosis, the miniature direct-on-blood PCR nucleic acid lateral flow immunoassay (mini-dbPCR-NALFIA) has been developed as an innovative molecular tool to directly test blood samples and uses a simple lateral flow strip for result read-out.

**METHODOLOGY/PRINCIPLE FINDINGS:** The performance of mini-dbPCR-NALFIA for diagnosis of VL in blood was assessed in a laboratory evaluation. The limit of detection, determined by testing blood dilution series of cultured *Leishmania* promastigotes, was 650 and 500 parasites per mL of blood for *Leishmania donovani* and *Leishmania infantum*, respectively. The diagnostic accuracy of mini-dbPCR-NALFIA was estimated for a set of 146 blood samples from VL-suspected patients from Spain, and for a sample set from 58 VL patients, 10 malaria patients and 68 healthy controls from Ethiopia and Kenya. The sensitivity and specificity of mini-dbPCR-NALFIA in the Spanish samples were 95.8% (95% CI, 88.3% - 99.1%) and 97.2% (95% CI, 90.3% - 99.7%), respectively, compared to a nested PCR reference standard. In the East African samples, the assay had a pooled sensitivity of 87.9% (95% CI, 76.7% - 95.0%) and pooled specificity of 100% (95% CI, 95.4% - 100%) using quantitative PCR as reference standard.

**CONCLUSIONS/SIGNIFICANCE**: Mini-dbPCR-NALFIA is a promising tool for simplified molecular diagnosis of VL and follow-up of treated patients in blood samples. Future studies should evaluate its use in endemic, resource-limited settings, where mini-dbPCR-NALFIA may provide a more reliable and versatile alternative to rK39 ICTs.

**Keywords:** visceral leishmaniasis, *Leishmania*, simplified molecular diagnostics, miniPCR, Nucleic Acid Lateral Flow Immunoassay

**Author summary**

Visceral leishmaniasis (VL) is a severe and lethal infectious disease caused by the unicellular parasites of the *Leishmania donovani* complex. The disease is prevalent in the Indian subcontinent, remote areas in East Africa as well as the Mediterranean. As VL is fatal when left untreated, early and confirmatory laboratory diagnosis is crucial for optimum treatment outcome. However, currently available serological assays, such as the rK39 rapid diagnostic test, fall short in terms of sensitivity and cannot be used for detection of relapsing infections. Therefore, we designed and evaluated an innovative and user-friendly diagnostic assay called mini-dbPCR-NALFIA, which is based on the direct detection of *Leishmania* kinetoplast DNA in human blood samples. Using cultured *Leishmania* parasites diluted in blood, we found that mini-dbPCR-NALFIA can detect down to 500 parasites per milliliter of blood, which will facilitate the diagnosis of patients with low parasite densities in point-of-care units. Furthermore, we assessed the accuracy of mini-dbPCR-NALFIA using a set of Spanish and East-African samples from VL patients and control groups. In the Spanish samples, sensitivity of mini-dbPCR-NALFIA was very high and somewhat better than in the East-African samples. The specificity of mini-dbPCR-NALFIA was excellent in both sample sets. In conclusion, considering its robust performance and simplicity, the mini-dbPCR-NALFIA is a promising diagnostic assay that has the potential to improve VL diagnosis and follow-up of treated patients, especially in resource-limited situations.

**Introduction**

Visceral leishmaniasis (VL), or “kala-azar”, is a severe parasitic disease caused an infection with the protozoan parasites *Leishmania donovani* or *Leishmania infantum*. These unicellular parasites are transmitted by blood-feeding female sandflies and infect phagocytes of the human host’s reticuloendothelial system (1). VL has a global estimated incidence of 50,000 to 90,000 cases per year, although true numbers are likely to be higher due to inadequate case recognition, lack of proper surveillance and appropriate reporting (2). Some of the most important endemic foci of VL are found in poor and remote regions of East Africa, where *L. donovani* is the causative species (3). Here, VL morbidity and mortality have a debilitating impact on affected populations (4-7). In Europe, zoonotic transmission of *L. infantum* occurs in the Mediterranean region, particularly in Spain and the Balkan peninsula (8). Although the relative contribution of European cases to the global VL burden is small, local outbreaks of the disease, such as the large epidemic in Madrid (Spain) between 2009 and 2018, or the outbreak in Bologna Province (Italy), may still pose a significant health problem (9, 10).

In clinical infections, VL presents as a serious illness with chronic fever, weight loss, hepatosplenomegaly and pancytopenia. When left untreated, the disease will be fatal in over 95% of the patients (2). Accurate diagnosis is therefore key in the timely detection and management of VL. The gold standard for VL diagnosis is the microscopic demonstration of *Leishmania* amastigotes in aspirates from infected tissues (11). Examination of splenic aspirates is the most sensitive, but its (incorrect) sampling may cause fatal hemorrhages, especially in thrombocytopenic and malnourished patients (12, 13). Alternatively, bone marrow or lymph node specimens may be used, but their sensitivity is often moderate to poor (13-15). As a result, aspirate microscopy is generally unsuited as primary diagnostic in rural settings, and the diagnosis is mostly based on serological assays as an alternative. In particular, easy-to-use and affordable rapid immunochromatographic tests (ICTs) detecting antibodies against *Leishmania* recombinant 39-amino acid repeat antigen (rK39) in capillary blood have been implemented as first-line VL diagnostic in many endemic areas (16, 17). Nevertheless, rK39 ICTs have a number of important shortcomings. For example, they are unsuited for monitoring VL treatment response because *Leishmania* antibody levels generally remain elevated in blood long after initial infection and treatment, which also limits the use of rK39 ICTs for diagnosing relapses of the disease (18, 19). Furthermore, serological tests show reduced sensitivity in immunocompromised VL patients, who often have impaired antibody production (20-22). This is especially a problem in East Africa, where VL-HIV co-infections can be common and sensitivity of rK39 ICT is significantly lower (23).

Molecular techniques, such as polymerase chain reaction (PCR), have several advantages over microscopic and serological methods for VL diagnosis. For example, they generally have high sensitivity and specificity (24, 25). Moreover, PCR can also be used to monitor treatment response and detect relapses in patients who have been treated for VL (26). These assets have led to the implementation of PCR-based assays as reference test for VL diagnosis in many European countries. However, application of molecular techniques in endemic areas of East Africa is hampered by their complexity, costs and the requirement of advanced laboratory infrastructure, including sophisticated equipment for amplification and result read-out.

In recent years, several simplified molecular techniques have been developed to overcome these practical limitations of conventional PCR. One example is the loop-mediated isothermal amplification (LAMP), an isothermal DNA amplification assay for which the results can be read out with the naked eye thanks to fluorescence or turbidity caused by reaction by-products. A recent meta-analysis showed that, compared to aspirate microscopy, LAMP has a sensitivity of 93.8% and specificity of 97.2% for detecting VL infections in blood samples (28). Notwithstanding these promising results, a major drawback of LAMP remains the need of DNA extraction from the patient blood sample prior to testing, which complicates point-of-care implementation of LAMP due to additional equipment requirements and increased risk of contamination (28). Additionally, current LAMP protocols are not suited for multiplex target detection and therefore do not include amplification of a DNA extraction control.

An alternative that does not suffer from these limitations is the miniature direct-on-blood PCR nucleic acid lateral flow immunoassay (mini-dbPCR-NALFIA) (27, 28). This PCR-based method is applied directly on EDTA blood without the requirement of DNA purification. The PCR is run on a miniPCR device, a portable miniature thermal cycler which is easily programmed through a smartphone application. By using 5’-labelled primers, the PCR products will carry a tag that will allow their detection with NALFIA, a dip strip with a fast and straight-forward read-out. A NALFIA strip has two antibody test lines, each directed against a unique amplicon tag, meaning that mini-dbPCR-NALFIA is suited for simultaneous detection of two different PCR targets. Its user-friendliness and minimal needs for laboratory equipment make the mini-dbPCR-NALFIA a strong candidate for molecular diagnosis of VL in rural endemic settings. This study aimed to evaluate the performance of mini-dbPCR-NALFIA for detection of VL infection in blood samples from East Africa and Spain, compared to rK39 ICT as currently implemented serological test and quantitative PCR (qPCR) or *Leishmania* nested PCR (LnPCR) as reference.

**Methods**

**Ethical clearance**

This study involved testing of clinical blood specimens from three different VL-endemic countries: Spain, Kenya and Ethiopia. For the use of clinical blood samples from the WHO Collaborating Centre for Leishmaniasis (WHO-CCL) at the Carlos III Health Institute (ISCIII) in Madrid, Spain (registered at the National Biobank Register, Section Collections, Spain, reference ID: C.0000898), ethical clearance was obtained (ref. APR12–65 and APR14-64). For the collection and use of the blood samples from Kenya, ethical permission was obtained at the Amref Health Africa Ethics and Scientific Review Committee (ref. ESRC P1196/2022) as well as the National Association for Science, Technology and Innovation (ref. NACOSTI/P/22/18832) in Kenya. For the Ethiopian blood samples, the study received approval from the Institution Research Review Board of the College of Health Sciences, Mekelle University, Ethiopia (ref. ERC# -1102/2017).

Before sample collection, written informed consent and assent were obtained from all participants and their respective parents or guardians.

Furthermore, samples from healthy Dutch blood donors that were deposited in a pre-established Biobank at the Laboratory for Experimental Parasitology at the Academic Medical Centre (AMC, Amsterdam, The Netherlands) and collected in accordance with the Dutch Medical Research involving Human Subjects Act (ref. BWBR0009408), were used to determine the limit of detection of the mini-dbPCR-NALFIA. All samples used in this study were anonymized.

**Mini-dbPCR-NALFIA**

The mini-dbPCR-NALFIA assay was originally developed for molecular diagnosis of malaria in resource-limited settings (27, 28). Its principle is described in detail elsewhere (28). For the current study, the dbPCR reagent mix and PCR run protocol were adjusted and optimised for diagnosis of VL in whole blood, through detection of *Leishmania* kinetoplast DNA (kDNA) with a human glyceraldehyde-3-phosphate dehydrogenase (GAPDH) gene target as an internal amplification control. Briefly, 2.5 µL of EDTA anti-coagulated venous blood was lysed by heating at 98°C for 10 minutes on a miniPCR mini16 (miniPCR bio, Massachusetts, USA). After lysis, 22.5 µL dbPCR reagent mix, consisting of 12.5 µL MyTaq Blood-PCR Mix (Meridian Bioscience, Cincinatti, USA), labelled forward and reverse primers for a kDNA target and GAPDH target (Eurogentec, Liège, Belgium) (**Table 1**) and nuclease-free sterile water, was added to the blood template. The used kDNA and GAPDH primers had been designed previously (29, 30).

Next, direct on blood amplification was performed in a mini16 thermal cycler with the following protocol: 3 minutes at 95°C; 30 cycles of 15 seconds at 95°C, 30 seconds at 58°C and 45 seconds at 72°C; and a final 2 minutes at 72°C. A NALFIA dipstick (Abingdon Health, York, UK) was used for read-out and placed in a mixture of 10 µL PCR product and 140 µL running buffer. During a 10-minute incubation, the labelled PCR amplicons flow over the NALFIA strip. They first pass the conjugate pad, where neutravidin-labelled carbon particles bind to the amplicons’ biotin label. Next, the kDNA and GAPDH amplicons are captured by an anti-Dig and anti-FAM antibody line, respectively. Accumulation of captured amplicons resulted in the appearance of a visible test line (see **Fig 1**).

A blood sample is considered to be positive for VL by the presence of a visible test line directed against Dig-labelled kDNA amplicons. A test was considered valid when the kDNA and/or GAPDH line were positive, together with a positive flow control line.

**Table 1.** **Primer sequences, labels and final concentrations used in the direct-on-blood PCR.**

| **Primer** | **Sequence** | **5’ labelling** | **Final concentration (nM)** |
| --- | --- | --- | --- |
| kDNA Forward | 5' – TCCCAAACTTTTCTGGTCCT – 3' | Dig | 250 |
| kDNA Reverse | 5' – TTACACCAACCCCCAGTTTC – 3' | Biotin | 250 |
| Human GAPDH Forward | 5’ – GAAGGTGAAGGTCGGAGTC – 3’ | FAM | 150 |
| Human GAPDH Reverse | 5' – GAAGATGGTGATGGGATTTC – 3' | Biotin | 150 |

Dig: digoxigenin; FAM: fluorescein amidite.

**Fig 1. Possible NALFIA results after dbPCR for Leishmania kDNA and human GAPDH.** The strip has three potential lines that can appear: a kDNA test line (1), a human GAPDH test line (2) and a flow control line (FC). (A) Both the kDNA and GAPDH lines are positive, indicating this is a VL-positive test result. (B) The GAPDH line is absent, however the kDNA line is positive, meaning that the PCR amplification was not inhibited. This result is considered valid and positive for VL. (C) A negative kDNA result and a positive GAPDH line, thus this test is negative for VL. (D) Both kDNA and GAPDH lines are absent, meaning that the dbPCR reaction was not successful. Therefore, this test result is invalid. (E) The NALFIA has no test lines nor a flow control line, meaning that this NALFIA test is invalid.

**Limit of detection**

To determine the lowest *Leishmania* parasite density that is detectable with mini-dbPCR-NALFIA, serial dilutions of cultured *L. donovani* (strain WR352) and *L. infantum* (strain MHOM/TN/80/IPT-1) promastigotes in negative donor blood were tested 20 times. *L. donovani* and *L. infantum* promastigotes were grown in RPMI 1640 medium with 10% fetal calf serum and 2% penicillin/streptomycin (both 10,000 units per mL) at 25°C. Heat-inactivated culture isolates were used to prepare dilution series in EDTA blood from a healthy Dutch donor, with parasite densities ranging from 10^5^ to 10^1^ parasites per milliliter (p/mL) of blood. The limit of detection (LoD) was defined as the lowest parasite density that was detected with 95% confidence (i.e., at least 19 out of 20 tests).

**Sensitivity and specificity**

The diagnostic accuracy of mini-dbPCR-NALFIA for detection of VL in EDTA whole blood was evaluated by applying this assay on two sample sets from different VL-endemic regions, namely Spain and East Africa. Within East Africa, samples were collected in VL-endemic areas in Ethiopia and Kenya.

*Sample size determination*

To calculate the required sample size for the mini-dbPCR-NALFIA evaluations in Spain and East Africa, the following assumptions were taken into consideration: taking 80% power, 5% marginal error, expected sensitivity and specificity 90%, and expected disease prevalence 50%, the minimum sample size was 134, 67 VL-positive patients and 67 VL-negative subjects, as determined by the reference standard (31).

*Evaluation in Spanish samples*

The Spanish set consisted of 146 EDTA venous blood samples from VL-suspected patients from *L. infantum*-endemic areas in Spain, collected from 2019 to 2022 by the WHO-CCL at ISCIII in Madrid, Spain. Prior to this study, DNA had been extracted from 200 µL of all samples using the QIAamp DNA Mini Kit (QIAGEN, Hilden, Germany) following manufacturer instructions. Isolated DNA was eluted in 200 µL PCR-grade water, and the eluates were tested with LnPCR targeting the *Leishmania* 18s rRNA gene as described by Chicharro *et al* (31). This LnPCR is the diagnostic reference standard in WHO-CCL laboratory and has a LoD of 5 parasite equivalents (par-eq) per reaction (31). Additionally, all samples had been serologically tested with Kalazar Detect (InBios, Seattle, USA) rK39 ICT. For the purpose of this study, all samples were tested with mini-dbPCR-NALFIA at ISCIII, together with quantification of the parasite load of all LnPCR-positive samples with kDNA qPCR as follows: 1 µL of sample DNA eluate was added to 24 µL of qPCR reagent mix consisting of 12.50 µL 2x Rotor-Gene SYBR Green PCR Master Mix (QIAGEN, Hilden, Germany), kDNA forward and reverse primers (500 nM each) as described by Mary *et al.* (32), and DNase-free water. The qPCR was run on a Rotor-Gene Q (QIAGEN, Hilden, Germany) using the following protocol: 10 minutes at 95°C, followed by 40 cycles of 10 seconds at 95°C, 30 seconds at 60°C and 20 seconds at 72°C. A melting curve analysis was included to confirm specific target amplification. Each qPCR run included a standard curve of a 10-fold serial dilution of culture-extracted *L. infantum* genomes (JPCM5 strain, MCAN/ES/98/LLM-724) for quantification of the sample parasite densities. qPCR data analysis was performed using Rotor-Gene Q Software.

*Evaluation in East African samples*

The second set of samples comprised 136 EDTA venous blood samples that were collected in East Africa. 47 of these samples were collected in 2022 in West Pokot County, Kenya, a region that is endemic for anthroponotic transmission of *L. donovani* (33). A total of 33 samples was taken from febrile patients in the regional kala-azar treatment centre at the Kacheliba Sub-County Hospital. All were tested with IT LEISH (Bio-Rad, Hercules, USA) rK39 ICT at the time of sample collection. 22 were rK39 ICT-positive VL patients, 10 were confirmed with *Plasmodium falciparum* malaria infection using Giemsa-stained blood smear microscopy and were negative for rK39 ICT, and 1 patient was both VL-positive with rK39 ICT and had a positive *P. falciparum* blood slide. The remaining 14 samples came from healthy controls from local villages in West Pokot and all tested negative with rK39 ICT at the time of sample collection.

The remaining 88 EDTA venous blood samples came from both VL patients and endemic (Southern and Western Tigray, Zone 2 and 4 of the Afar region and Abergele-Sekota Woredas of Amhara region) and non-endemic (Mekelle city and neighboring rural areas) healthy controls from Ethiopia. VL diagnosis of the 29 clinically suspected patients was confirmed using IT LEISH rK39 ICT during sample collection. The groups of 29 endemic and 30 non-endemic controls did not have VL-associated clinical signs and symptoms; all were negative with rK39 ICT, except 2 samples from endemic subjects. The blood samples were obtained at the Ayder Comprehensive Speciated Hospital, Mekelle and Kahsay Abera Hospital, Humera, Western Tigray, north Ethiopia from July 2019 to October 2020.

The Kenyan and Ethiopian sample sets were tested with mini-dbPCR-NALFIA at AMC, Amsterdam, the Netherlands. qPCR targeting *Leishmania* kDNA was chosen as reference standard for these East-African samples, as this is a highly sensitive and specific technique for diagnosing VL infections (24, 25). For qPCR, DNA was isolated from 50 µL of the EDTA blood samples using automated extraction with the NucliSENS EMAG (bioMérieux, Marcy-l’Étoile, France). The isolated DNA was eluted in 25 µL EMAG elution buffer. Next, 1.25 µL of DNA eluate was used as input for the reference qPCR targeting *Leishmania* kDNA and human GAPDH as DNA extraction control. The PCR reagent master mix solution consisted of 6.25 µl of iTaq Universal Probes Master Mix (Bio-Rad, Hercules, USA), 3.85 µl of DNase-free water, 260 nM kDNA forward and reverse primers and 100 nM kDNA probe (developed by De Paiva-Cavalcanti *et al.* (29)), and 100 nM GAPDH forward and reverse primers and probe (developed by Hennig *et al.* (30)). The amplification was performed using Bio-Rad CFX96 real-time PCR which was programmed as follows: 10 minutes of UNG activation at 50°C, 5 minutes of initial denaturation at 95°C, and 40 cycles of 15 seconds of denaturation at 95°C and 45 seconds of annealing/elongation at 59°C. A Cq value < 36 was considered positive for kDNA and GAPDH. Each qPCR run included a standard curve of DNA extractions from a 10-fold serial dilution of *L. donovani* promastigote culture (strain WR352) diluted in blood, which was used to quantify the parasite densities in the tested samples. The qPCR data analysis was carried out using Bio-Rad CFX Maestro 2.0 software.

**Statistical analysis**

All diagnostic test results were entered and analysed in Stata Statistical Software (Version 15, StataCorp LLC). The sensitivity and specificity of mini-dbPCR-NALFIA and rK39 ICT as currently implemented diagnostic were first calculated separately for the sample sets from the three different countries. For the samples from Spain, LnPCR was used as reference standard, since this is the VL diagnostic reference test at the WHO-CCL at ISCIII. kDNA qPCR was used as reference for the samples from Ethiopia and Kenya. Since these countries are both endemic for *L. donovani*, the pooled sensitivity and specificity of mini-dbPCR-NALFIA in East Africa was also calculated. The Clopper-Pearson method was applied for calculating the 95% confidence intervals (CI) of all sensitivity and specificity estimates. Cohen’s κ was calculated to express the level of agreement beyond chance between mini-dbPCR-NALFIA and the results of the reference tests and rK39 ICT.

**Results**

**Limit of detection**

The LoD of mini-dbPCR-NALFIA for *L. donovani* was determined to be 650 promastigotes per mL of blood. For *L. infantum*, the detection limit was 500 promastigotes per mL.

**Sensitivity and specificity**

*Spanish sample set*

The diagnostic test results for all samples included in this study are summarized in **Table 2**. 73 of the 146 EDTA blood samples from Spain tested positive for *Leishmania* with LnPCR and had a median parasite density of 3.2 x 10^3^ par-eq/mL of blood (interquartile range (IQR), 1.6 x 10^3^ – 1.1 x 10^4^ par-eq/mL), as determined with qPCR. Of these, 69 had a positive kDNA test line result with mini-dbPCR-NALFIA, 3 were negative for kDNA with NALFIA and 1 was invalid due to a negative result for both the kDNA and GAPDH test lines. The NALFIA-negative samples had a parasite burden of 48, 113 and 991 par-eq/mL. Of the 73 samples with a negative LnPCR result, 70 were also negative for the kDNA NALFIA line, 2 samples showed a positive kDNA test line and 1 sample had an invalid NALFIA result.

Using the LnPCR as reference standard, and excluding the samples with an invalid NALFIA result from the analysis, the sensitivity and specificity of mini-dbPCR-NALFIA were calculated to be 95.8% (95% CI, 88.3% - 99.1%) and 97.2% (95% CI, 90.3% - 99.7%), respectively, while the sensitivity and specificity of rK39 ICT were 71.2% (95% CI, 59.4% - 81.2%) and 94.5% (95% CI, 86.6% - 98.5%), respectively (see **Table 3**). The level of agreement between mini-dbPCR-NALFIA and LnPCR was almost perfect (Cohen’s κ: 0.93), while the agreement between rK39 ICT and LnPCR was only substantial (Cohen’s κ: 0.66). There was a considerable number of 21 samples that were negative for rK39 ICT but tested positive with LnPCR. Mini-dbPCR-NALFIA and rK39 ICT had a moderate agreement (Cohen’s κ: 0.58).

*East African sample set*

In the Kenyan sample set, 22 of the 23 samples from Kacheliba Hospital patients with clinical signs of VL and a positive rK39 ICT result were also kDNA positive with qPCR, with a median parasite load of 2.4 x 10^4^ par-eq/mL (IQR, 1.2 x 10^4^ - 4.1 x 10^4^ par-eq/mL). 19 of the 22 qPCR-positive samples were positive with mini-dbPCR-NALFIA. This included a malaria patient with a qPCR-confirmed VL co-infection. The 3 qPCR-positive samples with a negative NALFIA result had parasite densities of 318, 795 and 883 par-eq/mL. There were no positive qPCR, NALFIA or rK39 ICT results for the remaining 10 malaria patient samples. All 15 samples from the healthy endemic controls from West Pokot were negative with mini-dbPCR-NALFIA, rK39 ICT and qPCR.

There were no invalid tests for the samples from Kenya. With the kDNA qPCR performed at AMC as the reference standard, the sensitivity of mini-dbPCR-NALFIA was estimated to be 86.4% (95% CI, 65.1% – 97.1%) while its specificity was 100% (95% CI, 86.7% – 100%). rK39 ICT sensitivity and specificity were 100% (95% CI, 84.6% – 100%) and 96.2% (95% CI, 80.4% - 99.9%) (see **Table 3**), using qPCR as reference. Mini-dbPCR-NALFIA results were in almost perfect agreement with the qPCR results (Cohen’s κ: 0.87) and rK39 ICT results (Cohen’s κ: 0.83).

Among the 88 Ethiopian EDTA samples included in this study, all 29 VL patients positive for rK39 ICT during enrollment were positive with both mini-dbPCR-NALFIA and qPCR testing at AMC (see **Table 2**). Their median parasite concentration was 1.23 x 10^4^ par-eq/mL of blood (IQR, 6.4 x 10^2^ – 4.1 x 10^4^ par-eq/mL). From the healthy endemic control group, 2 out of 29 were positive for rK39 ICT at the site of sample collection, and at AMC, 7 were positive for qPCR, of which 3 were also mini-dbPCR-NALFIA positive. The parasite load of the mini-dbPCR-NALFIA-negative but qPCR-positive endemic controls were 79, 202, 335 and 624 par-eq/ml of blood. On the other hand, all samples from the healthy non-endemic areas were neither mini-dbPCR-NALFIA nor qPCR positive. Using qPCR as the reference standard, the sensitivity and specificity of mini-dbPCR-NALFIA in the Ethiopian samples were found to be 88.9% (95% CI, 82.3% - 95.5%) and 100% (95% CI, 93.2% - 100%), respectively. The agreement between mini-dbPCR-NALFIA and qPCR was excellent, with a Cohen's κ value of 0.91 (see **Table 3**). Similarly, the agreement between rk39 ICT and qPCR was also excellent (κ: 0.90). Mini-dbPCR-NALFIA and rK39 ICT had an almost perfect agreement (κ: 0.88).

Combining the evaluation results for the Ethiopian and Kenyan samples, the pooled sensitivity and specificity of mini-dbPCR-NALFIA for VL diagnosis in East Africa were 87.9% (95% CI, 76.7% - 95.0%) and 100% (95% CI, 95.4% - 100%), respectively (see **Table 3**). rK39 ICT had a pooled sensitivity of 87.9% (95% CI, 78.8% - 96.1%) and a pooled specificity of 97.4% (95% CI, 91.0% - 99.7%) in East Africa.

**Table 2. Overview of the diagnostic test results for the samples included in this study.**

|  | | **Mini-dbPCR-NALFIA** | | **rK39 ICT** | |
| --- | --- | --- | --- | --- | --- |
|  |  | Positive | Negative | Positive | Negative |
| **Spain (N=144)** | | | | | |
| LnPCR (reference standard) | Positive | 69 | 3 | 52 | 21 |
|  | Negative | 2 | 70 | 4 | 69 |
| Total | | **71** | **73** | **56** | **90** |
| **Kenya (N=48)** | | | | | |
| qPCR (reference standard) | Positive | 19 | 3 | 22 | 0 |
|  | Negative | 0 | 26 | 1 | 25 |
| Total | | **19** | **29** | **23** | **25** |
| **Ethiopia (N=88)** | | | | | |
| qPCR (reference standard) | Positive | 32 | 4 | 30 | 6 |
|  | Negative | 0 | 52 | 1 | 51 |
| Total | | **32** | **56** | **31** | **57** |
| **East Africa (Kenya and Ethiopia pooled) (N=136)** | | | | | |
| qPCR (reference standard) | Positive | 51 | 7 | 52 | 6 |
|  | Negative | 0 | 78 | 2 | 76 |
| Total | | **51** | **85** | **54** | **82** |

**Table 3. Accuracy estimates and Cohen's kappa for mini-dbPCR-NALFIA and rK39 ICT, compared to a PCR-based reference standard.**

| Sample set | Reference standard | Test | Sensitivity (95% CI) | Specificity (95% CI) | κ (95% CI) |
| --- | --- | --- | --- | --- | --- |
| Spain | LnPCR | Mini-dbPCR-NALFIA | 95.8% (88.3% - 99.1%) | 97.2% (90.3% - 99.7%) | 0.93 (0.87 - 0.99) |
|  |  | rK39 ICT | 71.2% (59.4% - 81.2%) | 94.5% (86.6% - 98.5%) | 0.65 (0.53 - 0.77) |
| Kenya | qPCR | Mini-dbPCR-NALFIA | 86.4% (65.1% - 97.1%) | 100.0% (86.8% - 100%) | 0.87 (0.73 - 1.00) |
|  |  | rK39 ICT | 100% (84.6% – 100%) | 96.2% (80.4% - 99.9%) | 0.96 (0.88 - 1.00) |
| Ethiopia |  | Mini-dbPCR-NALFIA | 88.9% (73.9% - 96.9%) | 100.0% (93.2% - 100%) | 0.90 (0.70 - 1.00) |
|  |  | rK39 ICT | 83.3% (67.2% - 93.6%) | 98.1% (89.7% - 100%) | 0.83 (0.62 - 0.99) |
| East Africa  (Kenya +  Ethiopia) |  | Mini-dbPCR-NALFIA | 87.9% (76.7% - 95.0%) | 100% (95.4% - 100%) | 0.89 (0.82 - 0.97) |
|  |  | rK39 ICT | 87.9% (78.8% - 96.1%) | 97.4% (91.0% - 99.7%) | 0.88 (0.80 - 0.96) |

**Discussion**

Due to the shortcomings of the aspirate microscopy as primary diagnostic, diagnosis of VL in resource-limited settings is mostly relying on the demonstration of anti-*Leishmania* antibodies in patient blood with rK39 ICTs. However, these tests have suboptimal sensitivity and specificity in most VL-endemic countries, especially in East-Africa (16, 34, 35). In addition, they are not suitable for the follow-up of treated or immunosuppressed patients. As an alternative to rK39 ICT, mini-dbPCR-NALFIA is a simplified, rapid, and robust test that employs amplification of *Leishmania* kDNA and lateral flow immunochromatography read-out. This study has evaluated the sensitivity and specificity of mini-dbPCR-NALFIA to diagnose VL directly on blood.

The limit of detection (LoD) was first determined using cultured *Leishmania* strains. There was a slight difference in LoD for *L. infantum* and *L. donovani*, namely 500 and 650 parasites/mL of blood, respectively. The difference could be the result of different copy numbers of the kDNA target in the used strains. Indeed, kDNA abundance has been shown to vary between *Leishmania* species (32, 36). Given that the analytical LoD difference for *L. infantum* and *L. donovani* is relatively small, the diagnostic detection limit of mini-dbPCR-NALFIA for both species is likely to be comparable. As the majority of tested patient samples had a parasite density above the LoD, mini-dbPCR-NALFIA is sensitive enough to be used in rural setting or any point of care with limited resources. To further lower the detection limit, additional optimization of the assay could be considered.

In the Spanish samples, using LnPCR as a reference test, the mini-dbPCR-NALFIA showed excellent sensitivity (95.8%) and specificity (97.2%). The sensitivity of mini-dbPCR-NALFIA in East African samples was found to be slightly lower: 86.4% in Kenya and 88.9% in Ethiopia. This difference could be explained by a number of factors, such as genetic variation among the etiological agents and the highly sensitive qPCR reference standard used for the East African samples. The lower sensitivity in East Africa may have resulted from parasite densities close to the LoD of mini-dbPCR-NALFIA, found in a number of samples and leading to a false-negative NALFIA result. On the other hand, the specificity of mini-dbPCR-NALFIA in the East-Africa was higher (100%) than in Spanish samples (97.2%). This is consistent with the fact that sensitivity and specificity are inversely related; when sensitivity increases, the specificity tends to decrease, and vice versa (37).

In the present study, the sensitivity of rk39 ICT was also assessed. In Spain the sensitivity was considerably lower (71.2%) than for mini-dbPCR-NALFIA (95.8%). This is in line with the previously reported rK39 ICT sensitivity of 78.0% in Spain and highlights the inferior performance of this serological test compared to molecular techniques (38). As such, mini-dbPCR-NALFIA can be considered a more reliable alternative VL diagnostic test in Spain and other Mediterranean countries, especially at the primary health facilities such as small hospitals, health centers and health posts, which now frequently rely on the less sensitive rK39 ICT (31). Additionally, its use for simplified molecular diagnosis of canine leishmaniasis, a common infection in domestic dogs in the Mediterranean and a potential reservoir for zoonotic *L. infantum* transmission, could be a topic of further study (39). Furthermore, a separate evaluation of mini-dbPCR-NALFIA for detection of New World *L. infantum*, which is genetically different from the Old World *L. infantum* strains in the Mediterranean, is warranted to assess the assay’s diagnostic application in South America (40).

Compared to Spain, in this study rK39 ICT performed better in East Africa, with an overall sensitivity of 87.9%. Indeed, previous studies in Western Kenya and neighboring Uganda found sensitivities of 97% and 89% of the IT LEISH in this area, using splenic aspirate microscopy and the serological direct agglutination test as reference (41, 42), while a recent meta-analysis reported a pooled rK39 ICT sensitivity of 88.0% in Ethiopia (34). Overall, the accuracy of mini-dbPCR-NALFIA in East Africa is similar to that of rK39 ICT, as is also reflected by the comparable levels of agreement of both tests with the qPCR reference standard. If mini-dbPCR-NALFIA is to be implemented in this area as more sensitive alternative to rK39 ICTs, further optimization of the assay might be necessary in order to increase the sensitivity (currently 87.9%). Nonetheless, mini-dbPCR-NALFIA remains a promising tool due to its robust performance, its feasibility to be performed in remote resource-limited endemic areas and its potential to differentiate active, past and relapse infections, which is not possible with rK39 ICTs.

The strength of this study lies in the evaluation of the mini-dbPCR-NALFIA on sample sets from different epidemiological settings: in Spain, the responsible agent is *L. infantum*, a zoonotic parasite (43); in East Africa, VL is caused by anthroponotic transmission of *L. donovani*, with different strains of this species circulating in Ethiopia and Kenya (44, 45). The results demonstrate how mini-dbPCR-NALFIA shows good performance in all three different countries. The negative NALFIA results for the Kenyan samples from patients with malaria, another febrile protozoan infection which is often co-endemic with VL, further underline the specificity of the assay. Moreover, the mini-dbPCR-NALFIA performed well at both the laboratories of AMC and ISCIII, demonstrating its robustness.

While the number of samples from East Africa that was evaluated in this study was sufficient to make a precise estimation of the mini-dbPCR-NALFIA performance in this region, the relatively small sample sizes from the individual countries could obscure possible differences in the assay’s accuracy in Ethiopia and Kenya. Accurate per-country comparison of mini-dbPCR-NALFIA with rK39 ICT, of which performance can vary considerably between different geographical regions (16), was also hampered by the small sample sizes. Another limitation of this study was the use of different reference standards by the evaluations at AMC and ISCIII. Although both LnPCR and qPCR have been shown to be highly accurate methods for diagnosing VL (31, 46), it cannot be excluded that sample classification had been different if the same reference standard had been used in both evaluations.

In conclusion, mini-dbPCR-NALFIA is a simplified molecular diagnostic test for VL, with excellent performance for detecting *L. infantum* infections in patients from Spain, where it provides a more sensitive alternative to the underperforming rK39 ICT. In East Africa, where the use of traditional molecular methods is impeded by the limited resources in VL-endemic regions, the easy-to-use mini-dbPCR-NALFIA performed similarly to rK39 ICT, the current cornerstone of VL diagnosis. Further improvement of mini-dbPCR-NALFIA should aim to lower its detection limit and thereby increase its sensitivity. Moreover, its application in situations where serological tests are unsuited, such as treatment monitoring and VL diagnosis in immunocompromised and relapsing patients, should be the topic of future studies.

**Acknowledgements**

We would like to express our acknowledgements to Mekelle University, College of Health Sciences and University of Amsterdam, Academic Medical Centre for their technical support during sample collection and laboratory analysis. We would like to thank the WHO Collaborating Center for Leishmaniasis at ISCIII in Madrid, Spain, led by Dr. Javier Moreno, for collaborating in this research and the support during the development and validation of mini-dbPCR-NALFIA for VL. Our gratitude also goes to the clinical staff at the Kacheliba Sub-County Hospital in Kenya and at the Ayder Comprehensive Speciated Hospital, Mekelle and Kahsay Abera Hospital, Humera, in Ethiopia for the collection of the East-African patient and control samples. Last but not least, we want to thank all the study participants for their voluntary participation in the study.

**Conflict of interest**

The authors do not have any conflict of interest to declare.

**Consent to publish**

All human subjects from which the samples used in this study were derived gave their written informed consent to publish the outcomes of the analysis on their samples.

**Author contributions**

NvD was responsible the study conceptualization and for the initial development of the assay. He coordinated the assay optimization and laboratory evaluation as well as the sample collection in Kenya, performed the testing of the Kenyan samples and, together with DGH, drafted the manuscript. DGH also performed all laboratory testing of the Ethiopian samples at AMC. DMH and SA assisted in the initial design and optimization of the mini-dbPCR-NALFIA for VL, and DMH performed the testing of the Spanish blood samples with mini-dbPCR-NALFIA at ISCIII. This sample set was assembled and tested with LnPCR by CC, while JCS designed and performed the qPCR assay at ISCIII. EC gave input during the design of the evaluation study, provided the Spanish sample set and coordinated all study procedures at ISCIII. DK and EA were responsible for the collection of samples at the Kacheliba Sub-County Hospital in Kenya. DW secured project funding and made the arrangements for sample collection in Ethiopia. HS was involved in study conceptualization, provided input and feedback during the assay development and evaluation and critically read the manuscript. All authors read and approved the final manuscript.

**Data availability**

The datasets used and/or analyzed during the current study are available upon reasonable request directed to either the corresponding author Norbert van Dijk or to Dr. Rens Zonneveld, both affiliated with the Amsterdam University Medical Centres, Academic Medical Centre at the University of Amsterdam, Department of Medical Microbiology and Infection Prevention, Amsterdam, the Netherlands. Data will be made available in an anonymized manner. Dr. Zonneveld’s contact details are as follows: Dr. R. Zonneveld, Academic Medical Centre, Department of Medical Microbiology and Infection Prevention, room L1-117, Meibergdreef 9, 1105AZ, Amsterdam, the Netherlands. Email address: [r.zonneveld@amsterdamumc.nl](mailto:r.zonneveld@amsterdamumc.nl).

**Funding**

This study was part of the EDCTP2 programme supported by the European Union: “Evaluation of the LAMP & db-PCR-NALFIA for the Diagnosis and/or as Test-of-Cure in Patients with Visceral Leishmaniasis in Ethiopia”; acronym “EvaLAMP & db-NALFIA“; grant number TMA2016SF- 1437; grant holder: DW; <http://www.edctp.org/projects-2/> . The funder did not have any input in study design, sample/data collection and analysis, manuscript preparation and publication.

**References**

1.	Poulaki A, Piperaki E-T, Voulgarelis M. Effects of Visceralising Leishmania on the Spleen, Liver, and Bone Marrow: A Pathophysiological Perspective. Microorganisms. 2021;9(4):759.

2.	Leishmaniasis: World Health Organization; 2023 [updated 12-01-2023. Available from: <https://www.who.int/news-room/fact-sheets/detail/leishmaniasis#:~:text=Visceral%20leishmaniasis%20(VL)%2C%20also,East%20Africa%20and%20in%20India>.

3.	Alvar J, den Boer M, Dagne DA. Towards the elimination of visceral leishmaniasis as a public health problem in east Africa: reflections on an enhanced control strategy and a call for action. Lancet Glob Health. 2021;9(12):e1763-e9.

4.	Meheus F, Abuzaid AA, Baltussen R, Younis BM, Balasegaram M, Khalil EAG, et al. The economic burden of visceral leishmaniasis in Sudan: an assessment of provider and household costs. Am J Trop Med Hyg. 2013;89(6):1146-53.

5.	Al-Salem W, Herricks JR, Hotez PJ. A review of visceral leishmaniasis during the conflict in South Sudan and the consequences for East African countries. Parasites & Vectors. 2016;9(1):460.

6.	Sunyoto T, Adam GK, Atia AM, Hamid Y, Babiker RA, Abdelrahman N, et al. "Kala-Azar is a Dishonest Disease": Community Perspectives on Access Barriers to Visceral Leishmaniasis (Kala-Azar) Diagnosis and Care in Southern Gadarif, Sudan. Am J Trop Med Hyg. 2018;98(4):1091-101.

7.	Coulborn RM, Gebrehiwot TG, Schneider M, Gerstl S, Adera C, Herrero M, et al. Barriers to access to visceral leishmaniasis diagnosis and care among seasonal mobile workers in Western Tigray, Northern Ethiopia: A qualitative study. PLoS Negl Trop Dis. 2018;12(11):e0006778.

8.	Van der Auwera G, Davidsson L, Buffet P, Ruf M-T, Gramiccia M, Varani S, et al. Surveillance of leishmaniasis cases from 15 European centres, 2014 to 2019: a retrospective analysis. Eurosurveillance. 2022;27(4):2002028.

9.	Horrillo L, Castro A, Matía B, Molina L, García-Martínez J, Jaqueti J, et al. Clinical aspects of visceral leishmaniasis caused by L. infantum in adults. Ten years of experience of the largest outbreak in Europe: what have we learned? Parasit Vectors. 2019;12(1):359.

10.	Varani S, Cagarelli R, Melchionda F, Attard L, Salvadori C, Finarelli AC, et al. Ongoing outbreak of visceral leishmaniasis in Bologna Province, Italy, November 2012 to May 2013. Euro Surveill. 2013;18(29):20530.

11.	Burza S, Croft SL, Boelaert M. Leishmaniasis. The Lancet. 2018;392(10151):951-70.

12.	Thakur S, Joshi J, Kaur S. Leishmaniasis diagnosis: an update on the use of parasitological, immunological and molecular methods. Journal of Parasitic Diseases. 2020;44(2):253-72.

13.	Zijlstra EE, Ali MS, el-Hassan AM, el-Toum IA, Satti M, Ghalib HW, et al. Kala-azar: a comparative study of parasitological methods and the direct agglutination test in diagnosis. Trans R Soc Trop Med Hyg. 1992;86(5):505-7.

14.	Siddig M, Ghalib H, Shillington DC, Petersen EA. Visceral leishmaniasis in the Sudan: comparative parasitological methods of diagnosis. Trans R Soc Trop Med Hyg. 1988;82(1):66-8.

15.	Ho EA, Soong TH, Li Y. Comparative merits of sternum, spleen and liver punctures in the study of human visceral leishmaniasis. Trans R Soc Trop Med Hyg. 1948;41(5):629-36.

16.	Boelaert M, Verdonck K, Menten J, Sunyoto T, van Griensven J, Chappuis F, et al. Rapid tests for the diagnosis of visceral leishmaniasis in patients with suspected disease. Cochrane Database Syst Rev. 2014;2014(6):Cd009135.

17.	Mohebali M, Keshavarz H, Shirmohammad S, Akhoundi B, Borjian A, Hassanpour G, et al. The diagnostic accuracy of direct agglutination test for serodiagnosis of human visceral leishmaniasis: a systematic review with meta-analysis. BMC Infectious Diseases. 2020;20(1):946.

18.	Kip AE, Balasegaram M, Beijnen JH, Schellens JH, de Vries PJ, Dorlo TP. Systematic review of biomarkers to monitor therapeutic response in leishmaniasis. Antimicrob Agents Chemother. 2015;59(1):1-14.

19.	Zijlstra EE, Nur Y, Desjeux P, Khalil EA, El-Hassan AM, Groen J. Diagnosing visceral leishmaniasis with the recombinant K39 strip test: experience from the Sudan. Trop Med Int Health. 2001;6(2):108-13.

20.	Cota GF, de Sousa MR, Demarqui FN, Rabello A. The diagnostic accuracy of serologic and molecular methods for detecting visceral leishmaniasis in HIV infected patients: meta-analysis. PLoS Negl Trop Dis. 2012;6(5):e1665.

21.	Leveque MF, Battery E, Delaunay P, Lmimouni BE, Aoun K, L'Ollivier C, et al. Evaluation of six commercial kits for the serological diagnosis of Mediterranean visceral leishmaniasis. PLoS Negl Trop Dis. 2020;14(3):e0008139.

22.	ter Horst R, Tefera T, Assefa G, Ebrahim AZ, Davidson RN, Ritmeijer K. Field evaluation of rK39 test and direct agglutination test for diagnosis of visceral leishmaniasis in a population with high prevalence of human immunodeficiency virus in Ethiopia. Am J Trop Med Hyg. 2009;80(6):929-34.

23.	Diro E, Lynen L, Ritmeijer K, Boelaert M, Hailu A, van Griensven J. Visceral Leishmaniasis and HIV coinfection in East Africa. PLoS Negl Trop Dis. 2014;8(6):e2869.

24.	De Ruiter CM, Van Der Veer C, Leeflang MMG, Deborggraeve S, Lucas C, Adams ER. Molecular tools for diagnosis of visceral leishmaniasis: Systematic review and meta-analysis of diagnostic test accuracy. Journal of Clinical Microbiology. 2014;52(9):3147-55.

25.	Galluzzi L, Ceccarelli M, Diotallevi A, Menotta M, Magnani M. Real-time PCR applications for diagnosis of leishmaniasis. Parasites & Vectors. 2018;11(1):273.

26.	Verrest L, Kip AE, Musa AM, Schoone GJ, Schallig H, Mbui J, et al. Blood Parasite Load as an Early Marker to Predict Treatment Response in Visceral Leishmaniasis in Eastern Africa. Clin Infect Dis. 2021;73(5):775-82.

27.	Roth JM, de Bes L, Sawa P, Omweri G, Osoti V, Oberheitmann B, et al. Plasmodium Detection and Differentiation by Direct-on-Blood PCR Nucleic Acid Lateral Flow Immunoassay: Development, Validation, and Evaluation. J Mol Diagn. 2018;20(1):78-86.

28.	van Dijk NJ, Menting S, Wentink-Bonnema EMS, Broekhuizen-van Haaften PE, Withycombe E, Schallig HDFH, et al. Laboratory evaluation of the miniature direct-on-blood PCR nucleic acid lateral flow immunoassay (mini-dbPCR-NALFIA), a simplified molecular diagnostic test for Plasmodium. Malaria Journal. 2023;22(1):98.

29.	de Paiva Cavalcanti M, Felinto de Brito ME, de Souza WV, de Miranda Gomes Y, Abath FG. The development of a real-time PCR assay for the quantification of Leishmania infantum DNA in canine blood. Vet J. 2009;182(2):356-8.

30.	Hennig H, Luhm J, Hartwig D, Klüter H, Kirchner H. A novel RT-PCR for reliable and rapid HCV RNA screening of blood donations. Transfusion. 2001;41(9):1100-6.

31.	Chicharro C, Nieto J, Miguelanez S, Garcia E, Ortega S, Pena A, et al. Molecular Diagnosis of Leishmaniasis in Spain: Development and Validation of Ready-To-Use Gel-Form Nested and Real-Time PCRs To Detect Leishmania spp. Microbiol Spectr. 2023:e0335422.

32.	Mary C, Faraut F, Lascombe L, Dumon H. Quantification of Leishmania infantum DNA by a real-time PCR assay with high sensitivity. J Clin Microbiol. 2004;42(11):5249-55.

33.	Mueller YK, Kolaczinski JH, Koech T, Lokwang P, Riongoita M, Velilla E, et al. Clinical epidemiology, diagnosis and treatment of visceral leishmaniasis in the Pokot endemic area of Uganda and Kenya. Am J Trop Med Hyg. 2014;90(1):33-9.

34.	Hagos DG, Schallig HDFH, Kiros YK, Abdulkadir M, Wolday D. Performance of rapid rk39 tests for the diagnosis of visceral leishmaniasis in Ethiopia: a systematic review and meta-analysis. BMC Infectious Diseases. 2021;21(1):1166.

35.	Abass E, Kang C, Martinkovic F, Semião-Santos SJ, Sundar S, Walden P, et al. Heterogeneity of Leishmania donovani parasites complicates diagnosis of visceral leishmaniasis: comparison of different serological tests in three endemic regions. PLoS One. 2015;10(3):e0116408.

36.	Weirather JL, Jeronimo SM, Gautam S, Sundar S, Kang M, Kurtz MA, et al. Serial quantitative PCR assay for detection, species discrimination, and quantification of Leishmania spp. in human samples. J Clin Microbiol. 2011;49(11):3892-904.

37.	Shreffler J, Huecker MR. Diagnostic testing accuracy: Sensitivity, specificity, predictive values and likelihood ratios. 2020.

38.	Bangert M, Flores-Chávez MD, Llanes-Acevedo IP, Arcones C, Chicharro C, García E, et al. Validation of rK39 immunochromatographic test and direct agglutination test for the diagnosis of Mediterranean visceral leishmaniasis in Spain. PLOS Neglected Tropical Diseases. 2018;12(3):e0006277.

39.	Morales-Yuste M, Martín-Sánchez J, Corpas-Lopez V. Canine Leishmaniasis: Update on Epidemiology, Diagnosis, Treatment, and Prevention. Veterinary Sciences. 2022;9(8):387.

40.	Alvarenga JS, Ligeiro CM, Gontijo CM, Cortes S, Campino L, Vago AR, et al. KDNA genetic signatures obtained by LSSP-PCR analysis of Leishmania (Leishmania) infantum isolated from the new and the old world. PLoS One. 2012;7(8):e43363.

41.	Chappuis F, Mueller Y, Nguimfack A, Rwakimari JB, Couffignal S, Boelaert M, et al. Diagnostic accuracy of two rK39 antigen-based dipsticks and the formol gel test for rapid diagnosis of visceral leishmaniasis in northeastern Uganda. J Clin Microbiol. 2005;43(12):5973-7.

42.	Mbui J, Wasunna M, Balasegaram M, Laussermayer A, Juma R, Njenga SN, et al. Validation of two rapid diagnostic tests for visceral leishmaniasis in Kenya. PLoS Negl Trop Dis. 2013;7(9):e2441.

43.	Alvar J, Cañavate C, Molina R, Moreno J, Nieto J. Canine leishmaniasis. Adv Parasitol. 2004;57:1-88.

44.	Zackay A, Nasereddin A, Takele Y, Tadesse D, Hailu W, Hurissa Z, et al. Polymorphism in the HASPB repeat region of East African Leishmania donovani strains. PLoS Negl Trop Dis. 2013;7(1):e2031.

45.	Zackay A, Cotton JA, Sanders M, Hailu A, Nasereddin A, Warburg A, et al. Genome wide comparison of Ethiopian Leishmania donovani strains reveals differences potentially related to parasite survival. PLoS Genet. 2018;14(1):e1007133.

46.	Adams ER, Schoone G, Versteeg I, Gomez MA, Diro E, Mori Y, et al. Development and Evaluation of a Novel Loop-Mediated Isothermal Amplification Assay for Diagnosis of Cutaneous and Visceral Leishmaniasis. J Clin Microbiol. 2018;56(7).
